# The Innervation of the Heart

**Published:** 1875-04-15

**Authors:** G. See


### THE INNERVATION OF THE HEART AND THE CARDIAC MEDICAMENTS.

By Professor G. See.

*Translated from La France Medicale*, 1874, *Nos.* 39, 43 *and* 57, *by H. M. Bannister, M. D.*

THERE is no subject at once more interesting and more complex than that of the innervation of the heart. This study, difficult as it may appear to you, is yet absolutely necessary, in order that you may understand the importance and the mode of action of the agents which I have called the cardiac medicaments.

As regards its structure, the heart is a striated muscle, but it differs from the other muscles of the life of relation in the fact that it possesses in its substance its own centres of innervation and of action. This peculiar character merits a special study of the medicines that affect it, and only this consideration justifies the autonomy of the therapeutic group of which I propose to treat.

As a muscle, that is, as a motor organ, the heart is controlled by two principal groups of medicines, namely:

1. Those which paralyze the motor nerves (curare, calabar bean, hemlock, aconite).

2. Those which paralyze the muscular system (salts of potash, veratria).

3. Farther, the heart possesses a system of irrigating canals which do not play a merely passive part. The vessels of the heart itself, the coronary arteries, possess an active attribute, that of contractility, which itself is under the control of the vaso-motor nervous system; therefore, any agent exercising a paralyzing influence on the vascular nerves may modify in a special manner the central organ of the circulation. Therefore, a third group of agents, the vascular medicines (belladonna, bromide of potassium, ergot, nicotine, etc.), may be considered as indirectly acting upon the heart itself.

4. Finally, there exists a last group of agents which appear to have an elective action on the heart, and which, for this reason, merit the title of direct or true cardiac medicaments, that I have given them.

Before studying the mode of action of this latter class, I think it may be advantageous to review the general subject of the physiology of the heart in regard to those points on which we have the most concise and positive information.

The innervation of the heart comprehends three centres of action:

1. The first of these is situated in the heart itself and constitutes its *automotor centre* or autonome. It is formed by little nervous ganglia situated in the sinus of the vena cava and in the walls of the left ventricle.

These ganglia contain in themselves the whole motor principle of the heart. There are those who have, in recent times, gone so far as to deny absolutely any other motor. Legallois was the first, at the beginning of the present century, to give out the opinion that the spinal cord might act upon the heart, and that there should be for this organ other auxiliary nervous centres. The means employed by Legallois to demonstrate the existence of these auxiliary centres were very primitive. His theory was, nevertheless, the correct one, notwithstanding the fact that Magendie and Claude Bernard disputed it. It belonged to a young German physiologist, Von Bezold, to place beyond any doubt the facts suggested by Legallois, and to demonstrate in an irrefutable manner the action which the cord exercises upon the heart. Von Bezold, in his beautiful experiments on the cardiac poisons, divided the nerves which go to the heart and preserved only a filament, starting from the grand sympathetic between the two thoracic ganglia; he observed:

1. That the pulsations of the heart became much less frequent.

2. That the vascular tension became greater.

3. That the issue of the blood from the arteries became more abundant and more forcible.

The cord, therefore, contains an auxiliary centre of action exercising its influence on the heart. But, by what way does the cord act on the heart? Nervous fibres leave the cord, passing by way of the thoracic nerve, according to some, by the pneumogastric, according to others.

Outside of this auxiliary spinal centre, there exists still another that acts indirectly on the heart—the *vasculo-motor* or *vasculo-nervous* centre. The vaso-motor nerves leave the spinal axis with the motor roots of the second cervical pair, and apparently have their origin in the upper part of the cord, and probably in the medulla. When this centre is affected it produces, as it were, a vascular paresis, characterized by a dilatation of the vessels and a lowering of the arterial tension. Thiry and Ludwig, in cutting the thoracic nerve, have observed the same phenomenon that Von Bezold noted in his experiment. After the section of the cord and of the cervical sympathetic, they noticed a notable lowering of the arterial pressure, and a diminution in the number of the pulsations. This last result, although contrary to the assertions of Professor Marey, ought to be considered as real and well-founded.

From these short physiological premises it results, therefore, that there are three cardiac centres of action:

*a*. An automotor centre (intracardiac ganglia).

*b*. An auxiliary spinal centre (spinal cord).

*c*. An indirect centre (vaso-motor nerves).

But these three centres are not alone sufficient to produce the regular play of this organ. There is still needed, in order to insure its marvelous harmony, a regulating agent, a sort of moderator that can prevent the troublesome effects of the influences we call moral, which singularly modify the contractions of the heart.

This regulator is the pneumogastric nerve, which seems to act in opposition to all the other nerves. In fact, we know that when we divide a motor nerve we immediately paralyze the muscles to which it goes, but when we cut the pneumogastric nerve we accelerate, as it were, the pulsations of the heart, instead of causing them to cease. The same nerve, when excited, instead of augmenting the action of the cardiac muscles, has the effect, on the contrary, to suspend its action.

The pneumogastric dominates, so to speak, over all the innervation of the heart; it is more powerful than the automotor centres; it governs, directs, arrests or counteracts them. The whole is subjected to its control; it is a ruler, a despot; or rather, it plays the same part as the curb or governor in the steam engine, as I have, following this analogy, already proposed for it the designation of governor of the heart, a name universally adopted at the present day. This depressing, or rather, suspensive action of the pneumogastric, admitted by the majority of physiologists, has nevertheless been denied by Prof. Schiff, and more recently by M. Onimus. Both refuse to admit the action of the vagus as a nerve of arrest, without denying altogether the retardation of the heart produced by the excitation of this nerve; but they seek to explain this effect, the one by the intensity of the excitation of the currents employed, the other by the action of related currents.

Besides the pneumogastric nerve, there is a second system of arrest, situated in the heart itself. There is, in fact, in that organ a nervous ganglia acting in a directly contrary manner to the others (the automotor centres), that I mentioned before. The physiological action of this inhibitory ganglion has been recently revealed to us by the singular properties of a new agent, studied by M. Prevost, of Geneva; I refer to muscarine. When the heart of an animal is taken from the chest, as is well known, it continues to pulsate for a few seconds, although separated from its nerves and nervous centres, on account of the nerve power it receives from its little inherent and automotor ganglia. But muscarine arrests the movements of the heart, when thus taken from the chest and still palpitating. This effect can only be explained by the action that muscarine exercises on the centre of arrest situated in the heart itself.

What is the connection between this intracardiac centre of arrest with the pneumogastric? In the actual condition of science it is difficult to answer this query, and it seems to us advisable, notwithstanding the ingenious hypotheses that have been proposed, to leave the question still in suspense.

The two systems of arrest I have described are not the only ones that control the heart; there is, in addition, an intimate correlation between the contractions of this organ and the cardiac circulation itself. It appears that everything connected with the heart has its centres of action and of arrest.

The vaso-motor system of the heart is provided with a special nerve, discovered by Ludwig and the brothers Cyon. This discovery obtained for the two last named physiologists the grand prize in experimental physiology decreed by the Academy of Sciences in 1867. The nerve of Cyon ordinarily rises by one root from the trunk of the pneumogastric, and the other from the superior laryngeal. It is separate in the rabbit and the dog, but in man is included with the trunk of the vagus nerve.

Starting from its origin in the upper part of the neck, the nerve of Cyon descends along the carotid artery by the side of the cervical filament of the sympathetic, which it accompanies without uniting with it. Reaching the chest, it anastomoses with fibres from the first thoracic ganglion, and is soon lost in the substance of the heart, or better in the dense cellular tissue situated between the origins of the aorta and the pulmonary artery.

Such, in a few words, is the anatomical description of this singular nerve which plays so important a part in the circulation by its reflex action on the motor nerves of the blood-vessels.

For the purpose of experiment, we expose and divide it in the middle portion of the neck. While the electric excitation of the peripheral portion of the divided nerve produces no pain and has no effect on the manometric pressure or on the heart, the excitation of its superior and central portion is painful and causes a depression of five or six centimetres in the manometer applied to the carotid—in a word, an enormous diminution of pressure in all the vessels. The mechanism of this reflex action on the blood pressure is shown in first paralyzing the muscular system with curare and then cutting all the nerves that go to the heart. The lowering of the pressure remaining, in this case, the same, renders it certain that it is not through the muscular system nor through the heart that it is brought about. But if we perform the section of the splanchnic vaso-motor nerves, this vascular depression fails to appear, and therefore we have the secret of its mechanism. The excitation of this sensitive cardiac nerve reacts, therefore, exclusively on the vaso-motor nerves, producing a repletion of the heart and a diminution of the blood pressure. To the direct paralyzing influence of the pneumogastric on the heart is added also a paralyzing reflex influence of the sensitive or depressor nerve of the heart. The nerve of Cyon is therefore a moderator or depressor nerve — a nerve of arrest of the vascular centre.

*En resume*, there exist three principal centres of action for the heart, and three corresponding antagonistic centres constitute its system of arrest.

1. An automotor centre of action (intra-cardiac ganglia) and an intra-cardiac centre of arrest.

2. An auxiliary medullary centre (spinal cord) and a corresponding nerve of arrest (the pneumogastric nerve).

3. An indirect centre (vasculo-nervous) and a special depressor nerve of the vaso-motor system (nerve of Cyon).

The physiological premises I have just laid down were a needed preface to the study of the cardiac remedies. How, indeed, can we understand the mode of action of a medicine on the heart if we do not know beforehand the functional endowments with which that organ is provided? Would we not be continually exposed to error, if, guided only by a blind empiricism, we were ignorant of the manner in which the medicine we prescribe ought to act? It is not sufficient to know that the drug enfeebles the contractions of the heart and diminishes the frequency of the pulse, we ought yet to know how it produces these effects. Is it the cardiac fibre itself that it acts upon, or rather are the nerves of the heart first influenced by the agent? In this latter case we have still to inquire whether we have produced, for example, an excitation of the nerves of arrest or a paralysis of the automotor centres.

In order to reply to these and like queries, and to answer the questions that every physician, jealous of his art, should propose, the previous study of the mode of action of a medicine is absolutely indispensable, and consequently experimentation, "*in corpore vili*," is needful, and should serve as the basis of the therapeutic application.

But as we advance in this study, new difficulties present themselves before us. The physiological deductions and the experimental results are far from giving us all the elements of so complex a problem. Medicines are like men, and we may apply to them the celebrated saying of Pascal: "There are no two of them that resemble each other. The more we advance, the more are we able to establish the distinctions which separate them and the shades of difference between them." But it is for us to discover, at the same time, the analogies and the differences that present themselves to us in this general study of the cardiac remedies.

There are two kinds of cardiac medicines: the one acts all at once and directly upon the heart; digitalis is the type of this kind. All the effects of this agent are the result of its primal action on the heart. The other kind acts principally on the other organs, and their action on the heart is only consecutive and secondary. These are the indirect cardiac agents; such, for example, are calabar bean, aconite, veratria, etc.

Allow me to say a few words on these two last-named medicines, as regards the special point of view which now occupies our attention.

*Aconite, Aconitia.*—Aconite had its day of splendor in the last century, in the hands of Stoeck, who recommended its employment in acute articular rheumatism. After his death the remedy fell to the rank of the anti-nervines. It seems even to have almost fallen into oblivion, when more recently, the homœopaths on the one hand, and on the other, Dr. Schroff, in Germany, the author of a very interesting treatise on pharmacology, brought it again into notice, and physiology itself comes to its aid in securing for it the recognition it deserves.

All the facts concur to show that aconite is a poison to the heart. I pass in silence the various effects produced by it on sensibility and motility, for here we are only concerned with its action on the heart. Administered in a rather large dose, aconite causes a retardation of the cardiac contractions and a diminution in number of the pulse beats. This action is very prompt, and death ensues from failure of the heart, before the lungs are at all affected. But let us attempt to penetrate farther in the study of its action, and first of all we must point out the different results according to the dose employed. When we give aconitia in an almost infinitesimal dose, $\frac{1}{5}$ of a milligramme (=.003 gr.), we observe a very noticeable acceleration of the pulse and the circulation. This preliminary effect is of short duration, for this action of aconite on the centres of excitation of the heart is ephemeral and transient.

As soon as this first phase of excitement has passed, we observe a singular state of fatigue of the heart, which shows itself first by a weakness of the ventricles and then of the auricles. It appears that the heart (pardon the expression) can flap but one wing, and, in fact, it only beats with the auricles, and at last stops, in a sort of tetaniform contraction. One of the factors of the inhibitory system ought to be found super-excited in order to produce this effect. But what is this inhibitory system of the heart? According to what I have said before, the cause must be sought in the pneumogastric nerve or the ganglia at its termination. It is the curb that is over applied, and the arrest takes place; the poison takes effect on the vagus nerve, and the heart undergoes a tetaniform contraction.

The excitation of the suspensive or inhibitory system becomes weaker, and finally ceases, while the convulsive contraction still continues. At the moment when the inhibitory excitement is exhausted, how is it that the heart remains in its, as it were, tetanized state? It is because aconitia is a nervo-muscular poison; it acts not only on the nerves, but on the cardiac muscle itself. We may sum up the phases of aconite poisoning as follows: In the first place we observe an acceleration of the pulsations of the heart, due, without doubt, to an excitation of the automotor centre.

In the second stage, there is a retardation of the pulse and the cardiac contractions, probably produced by an excitation of the nerve of arrest (pneumogastric).

In the last stage, we have arrest in tetanic contraction by exhaustion and paralysis of the suspensory system, and finally inertia of the muscle and paralysis of the intra-cardiac motor system.

*Veratria.*—Veratria offers some analogies with aconite, but it presents numerous differences as regards the considerable modifications it produces in the arterial pressure. It is in virtue of this special property that it may be considered as one of the most powerful anti-pyretic agents.

We find in this case, as in that of aconitia, a preliminary stage, which is the more pronounced when the veratria has been given in very weak doses. When we give 1 milligramme (=.015 grs.) of veratria, we augment instead of diminishing the peril. In fact, instead of retarding the pulse by the use of a small dose, we only accelerate it. This is a new and striking proof of the importance of this study, and of the difficulties with which it bristles. Therefore, we ought to take special care lest we employ a timorous and dangerous moderation in the choice of the dose we administer; for that which may seem to be an excess of prudence, in reality is only a blind temerity; because the action of veratria in too minute doses may produce the most deplorable results.

Veratria should be employed in the dose of from six to twelve milligrammes (=.09 to .18 grs.), in order to produce really useful therapeutic effects; that is, a considerable retardation of the contractions of the heart. Little by little, this retardation continues, even until the inhibitory system loses its excitability and its power. The pneumogastric nerve becomes exhausted in its trunk much sooner than in its branches, as the following experiment will prove:

Take an animal, administer to it veratria; we then observe a notable slowing of the pulse; next cut the pneumogastric, and the pulse becomes quickened; excite the peripheral portion of the nerve, and the pulsations again immediately become slower. At last, after a certain time, the heart itself is affected, as we have shown in the case of aconitia, and the irritability of the cardiac fibre itself becomes extinct.

We have stated that veratria exercises a very interesting influence on the vascular pressure: When we give it in the dose of about ten milligrammes, we observe, simultaneously with the enfeeblement of the pulse, a considerable lowering of the blood pressure, a diminution of the contraction of the heart, and a manifest reduction of the temperature.

But if we recall what has been said in regard to the nerve of Cyon, we find an important application of these physiological results. We are aware that if we cut this nerve and irritate its central portion we cause a centripetal excitation that diminishes the pressure throughout the whole arterial system, and in particular in the mesenteric system.

This nerve, therefore, counteracts the working of the vasculo-motor centre. The pneumogastric is a centrifugal, and therefore a motor nerve. The excitation of its peripheral portion corresponds in its effects to the irritation of the central end of the nerve of Cyon.

In all the experiments performed on the pneumogastric, we may note, simultaneously with the slowing of the pulse caused by the excitation of this nerve, a diminution in the vascular pressure; but the synchronism between the two phenomena is not complete and absolute; the sphygmograph shows, in reality, a diminution of the arterial tension previous to the retardation of the pulse; the two phenomena are parallel, but not connected.

However it may be, we find the physiological action of the nerve of Cyon in the effects of veratria on the vascular tension, and see by this how useful the application of experimental physiology may be to the study of the action of drugs.

In a second phase of the action of veratria the lowering of the vascular pressure becomes still more pronounced, and after a certain time the vascular walls present an extreme depressibility; the vessels and the heart itself are so far paralyzed that further excitation is impossible. In this third period the heart is affected, and the individual dies in a state of extreme weakness, of profound collapse, as is observed in certain cases of pneumonia treated with veratria.

We should therefore bear these facts in mind, and not fail whenever we administer this remedy, to attend to auscultation, and to watch with the greatest care the state of the heart and pulse during the whole time we employ this remedy, generally so efficacious, sometimes so dangerous, and always so delicate and difficult to direct.

Our attention has so far been particularly given to two of the agents which deserve to be placed in the foremost rank among the indirect cardiac medicaments: aconite and veratria, the one paralyzing the motor nerves of the heart, and the other acting on its muscular fibre. By the side of these we may place curare, calabar bean, hemlock and nicotine in one group, while the salts of potassium should be placed in a second by the side of veratria. All these agents diminish the excitability of the inhibitory system of the heart, but they employ different means to arrive at that result.

1. The one kind paralyze the terminal extremities of the nerves, the pneumogastric in particular (conium, curare); both of these two poisons affect the motor nerves of the life of relation before they act on the heart itself.

2. Others, before suppressing the functions of the organ itself, excite its inhibitory system (first period of nicotine and calabar bean poisoning).

3. Within five years, two experimenters at Dorpat, Dr. Oscar Schmiedeberg and Richard Koppe, have noted the singular effects of a new alkaloid obtained from poisonous mushrooms, and in particular the agaricus muscarius, whence the name muscarine, which they have given to the extract, and which was the subject of a very recent communication to the Societe de Biologie by Dr. Prevost, of Geneva.

This young and talented physiologist has shown that by injecting muscarine into the veins of an animal, we arrest the heart in diastole. The action of this poison is so much the more curious since in such a condition the heart is not dead, and is even not paralyzed, for we can revive its contractions after long hours of inaction. Such an effect can therefore only depend on the irritation of a nerve of arrest. Is it the pneumogastric that is here in action? Evidently not, since the previous section of this nerve, in an animal poisoned with muscarine, does not hinder the effects of the poison. It must, therefore, be the intracardiac centre of arrest itself on which muscarine acts.

Physostigmia, applied to man, causes a more or less considerable retardation of the heart's pulsations; but we do not yet know with certainty its real mode of action. When we act on a muscarinized heart (if I may be excused the expression) with physostigmia, we break in upon the silence of that organ which, before arrested in diastole, now commences to pulsate. We may therefore conclude that physostigmia acts on the heart in manner contrary to that of muscarine. As to conia, it produces an effect upon the heart only very slowly. Indeed, hemlock, in this respect, like curare, paralyzes all the motor nerves before attacking the central organ of the circulation; it is very probable that this poison acts, in causing the stoppage of the heart, by exciting the pneumogastric nerve.

In opposition to these last-named agents, belladonna and its derivative atropia, paralyze the cardiac extremity of the vagus. Their effects are therefore altogether the reverse of the preceding. The acceleration of the heart pulse in this case can only be attributed to a paralysis of the pneumogastric, or of the intracardiac centre of arrest; in a word, this effect is the direct opposite to that of muscarine.

It would be very interesting to pursue this study, and to be able by this physiological method to recognize as many special medicines as there are functions or particular systems in the cardiac organ. Unfortunately, such an analysis is barely sketched, and we cannot present here more than a distant and imperfect outline.

There still remains to be considered an agent the most important, and at the same time the best known. I refer to digitalis. Its history seems altogether exhausted; it appears as if nothing new could be added after the numerous memoirs of which it has been the subject. Nevertheless, all has not been said that may be said on this interesting topic. Many lacunæ remain to be filled, many dark points to be cleared up, many obscurities to be done away with.

We rank digitalis among the direct-acting cardiac agents, but we should bear in mind that its mode of action is none the less very difficult to explain. Two different methods have been followed to resolve this difficult problem.

On the one hand, we may perform the experiments on ourselves in a very simple fashion. The homœopaths first adopted this primitive procedure. The celebrated Hahnemann first of all experimented on chamomilla. He noticed, after the ingestion of this medicine, which, nevertheless, seems very inoffensive, a feeling of strangulation, of cephalalgia, numbness in the lower limbs, hallucinations, and confused and strange ideas. (There is no doubt but that the experimenter had no need of taking chamomilla in order to experience such effects.) If I mention these first attempts in experiment, it is solely for the purpose of putting others on their guard against similar errors. But it is in this manner that the effects of digitalis have been studied by two patient but fanciful physicians, Sanders and Stadion. Let us see how they have described them. In the first few days that followed the ingestion of the drug, they noted nothing in particular, but about the sixth day nausea and anorexia appeared. From this time on the trial *in anima medicarum*, became, as we can easily understand, very much interfered with. The subjects of the experiment ceasing to eat, excreted no more urea, and the results became false and erroneous.

Stadion and Sanders, after the eleventh day of their experiment, fell into a state of extreme feebleness, which is easily comprehended after so long a period of involuntary fasting. One of them himself assured me that he was so weak as to be unable to maintain an erect position. But let us leave these courageous but mistaken investigators, whom I only mention in order to point out the imperfections of their method.

All those who have made themselves the subjects of their experiments ought to have kept the conditions the same both before and during their investigation; this is where they have failed. A few, however, have sought to ward off this source of error. Voit has carried on a very remarkable series of researches on the effects of foods and medicines, by ascertaining very accurately the state of the ingestions and that of the excretions, both before and during the experiments.

Dr. Mejoin, on his part, has not only weighed the quantity of food ingested in his experiments, but he has also indicated the quality. In taking fifteen hundred grammes of food, we are able to tell just how much carbon and nitrogen it contains.

Voit fixed the amount of the expenditures, that is, the excretions, in order to be able to make out the complete physiological balance, and it is only at that price that he is able to lay down any really scientific conclusions.

In short, all the experiments on digitalis have been made in the most imperfect and coarsest manner. We can see in what respects they are faulty: "The first are altered by the loss of appetite which inevitably ensues a few days after the ingestion of digitalis; while those made by the second method are rendered erroneous from the want of a previous establishment of the exact balance in the organism.

In the presence of these obstacles in the way of experimentation, the imperfections of the methods employed, and the want of exactitude in the results obtained, we may understand the difficulties with which this study is surrounded. I will now try to infuse a little order and light into this chaos.

I. *Effects of digitalis on the heart and blood-vessels.* In addition to the researches of Sanders and Stadion, I ought here to notice the interesting monographs of Bordier, Legroux, and Lelion, among the more important works undertaken in late years on this subject.

Sanders has stated that when he took a moderate dose of digitalis, he observed during the first few days a notable acceleration of the pulse. The fundamental action of the drug followed this initial effect, namely, a retardation of the pulse, but while this retardation existed the slightest cause produced again a quickening.

With a large dose, the retardation appears, says he, in three hours, and attains its maximum at the end of twenty-four hours; if we continue this dose for five or six days, the pulse becomes small, irregular and intermittent. If it is necessary to purchase the slackening of the pulse at the expense of an acceleration that lasts two or three days, we are in danger of making false steps. But this initial acceleration fortunately has not been noticed by all the observers, and particularly by the young experimenters whose names we have given above. They have obtained the retardation of the pulse at once, and in a very satisfactory manner.

What is the cause of this retardation?

In order to explain it one eminent physiologist has practiced experimenting with an artificial circulatory apparatus, in which the heart was represented by a hollow bulb of india rubber, tubes of the same material taking the place of the vessels. By the aid of this instrument, ingenious no doubt, but assuredly imperfect, he has been enabled, he states, to formulate a very curious law: "When the calibre of the discharging tubes is contracted, the india rubber bulb can empty itself only with difficulty. If, therefore, the heart drives out the blood it contains with difficulty, we ought to find an augmentation of the pressure in the vessels, and reciprocally the increase of the intravascular pressure produces the slowing of the pulse" (Marey). I give this proposition in Prof. Marey's own words, without comment on my own part.

*Experiments on Animals.* We will now examine the results furnished by physiological experiment. It is necessary that we should exercise a choice in the animals which we would submit to experimentation. Some resist the poisons which are fatal to us; others are violently affected by those which are without effect upon ourselves. With the exception of the Guinea pigs, all the herbivora are resistant to the effects of digitalis, and it is necessary to have recourse to very powerful doses in order to produce appreciable results. On the carnivora, on the other hand, digitalis acts in a very marked manner.

Let us take a small dose of digitalis. I do not speak of digitaline, which differs according to the mode of preparation (Nativelle, Homolle, and Quevenne); the powdered digitalis is the preferable preparation. If, therefore, we inject a small quantity of the infusion into an animal, we observe almost immediately a slowing of the pulse. Increase, then, the dose (five or ten centigrammes of the solution), and we obtain the directly contrary effect, the heart commences to gallop. If you wish to produce this acceleration, you have only to employ the strong dose at first. It is therefore useless to make an algebraic equation with the words digitalis and retardation of the heart. If we inject a decidedly toxic quantity, we arrest the heart in systole. Thus we see that whenever we employ digitalis, we find ourselves, as it were, between two fires: on the one side the acceleration of the pulse, and on the other the arrest of the heart's action through excessive retardation. * * * *

The first effect of digitalis is exercised on a special nervous apparatus, while its later action takes place on the cardiac muscular apparatus itself. By this rule, the different action of the drug, according to the dose employed, should be explained.

We have seen the effects of digitalis as regards the retardation of the pulse, but it yet remains to us to study its effects on the vascular pressure. It is admitted, as a general rule, that digitalis augments the blood pressure, but it seems to me needful to establish here an important distinction: When we administer the medicine in therapeutic doses, the slackening of the pulse and the augmentation of the pressure progress *pari passu;* but if we give a stronger dose we have first an increase of blood pressure, and shortly after a more or less considerable decrease of the same. If, on the other hand, we administer a toxic dose, we obtain a diminution of a quarter or a third in the intra-vascular pressure.

These singular effects of digitalis may be summed up in the following synoptical table:

I. WITH THE SMALL THERAPEUTIC DOSE.

1. Retardation of the pulse.
2. Increase of the intra-vascular pressure.

II. WITH LARGE DOSE.

1. Acceleration of the pulse, which may terminate in a retardation.

2. Decrease of blood pressure, perhaps changing to an increase.

III. WITH A POISONOUS DOSE.

1. Sudden slowing of the heart, which is attacked in its muscular substance.

2. Permanent lowering of the blood pressure.

Digitalis is a singular poison, distinguished from most of the others by what we may call its cumulative action; when the doses rapidly follow each other their effects accumulate. Very few drugs possess like cumulative action. Very small but repeated doses may result in a sudden explosion of its dangerous effects. We insist upon this point, since this drug, at the same time so wonderful and so terrible, ought never to be given without a previous knowledge of these possible results.

There is finally a last point to which it appears useful to give our attention: that is the change in the contractile force of the heart, and in the quality of the pulse. Under the influence of digitalis, while the heart beats slower, its pulsations become more energetic, and perhaps it is the increased force of the contractions that produces the elevation of the blood pressure.

On the other hand, the pulse becomes dicrotous, it beats double, and if we cast our eye on the sphygmographic trace of the radial artery, we perceive that this dicrotism is on the descending line. This descending line only returns to its point of departure every two or three pulsations; in short, it fails to reach it, and we have then two or three ventricular systoles following each other before the heart succeeds in accomplishing one diastole. We find sometimes in the human subject that the pulse becomes double or triple, in a word, polycrotous. We ought to carefully take note of this disturbance in the rhythm of the pulse, as this last phenomenon is very frequent; it is met with even in cases where the digitalis is given in therapeutic doses, and when these relatively insignificant doses are kept up during a certain time.

Inversely, we employ digitalis to cause the disappearance of similar phenomena of arythmy depending on a disease of the heart, and which offer a very striking resemblance to those caused by the drug in a healthy individual.

---

RELATIONS OF ATMOSPHERIC ELECTRICITY AND OZONE TO DISEASE.—Dr. Geo. M. Beard, *Popular Science Monthly*, in a valuable discussion of the above topic, gives the following practical suggestions:

1. Many nervous and other diseases, and very many nervous sensations, are perceptibly affected by changes in the quantity of electricity and ozone. After eliminating the factors of heat and cold, of moisture and dryness, of carbonic and nitric acid, of oxygen, pure and simple, there remains much that only ozone and electricity can well account for.

2. Not a few sensitive and impressible organizations experience variations of strength and debility of vigor, and malaise, that very well correspond to the variations in atmospheric electricity, or ozone, or both. From 8 to 12 A. M. is the golden time for brain work, and from 4 to 8 P. M. the silver period for all mental labor; during these two periods there is the greatest amount of ozone and electricity in the air.

3. Irregular disturbances in the electrical conditions of the atmosphere in storms, and especially in thunder storms, and in our climate north-east storms unquestionably affect the nervous system of impressible temperaments unpleasantly, and bring on or aggravate neuralgic, rheumatic and other pains, as well as incite mental distress and discouragement. Now, just before thunder storms, atmospheric electricity is apt to be negative.

